# Multimodal photoacoustic/ultrasonic imaging system: a promising imaging method for the evaluation of disease activity in rheumatoid arthritis

**DOI:** 10.1007/s00330-020-07353-z

**Published:** 2020-11-12

**Authors:** Chenyang Zhao, Qian Wang, Xixi Tao, Ming Wang, Chen Yu, Sirui Liu, Mengtao Li, Xinping Tian, Zhenhong Qi, Jianchu Li, Fang Yang, Lei Zhu, Xujin He, Xiaofeng Zeng, Yuxin Jiang, Meng Yang

**Affiliations:** 1 Department of Ultrasound, Peking Union Medical College Hospital, Chinese Academy of Medical Sciences and Peking Union Medical College, Shuaifuyuan No.1, Dongcheng District, Beijing, 100730 China; 2 Department of Rheumatology and Clinical Immunology, Peking Union Medical College Hospital, Chinese Academy of Medical Sciences and Peking Union Medical College, Key Laboratory of Rheumatology and Clinical Immunology, Ministry of Education, Beijing, China; 3 Shenzhen Mindray Bio-Medical Electronics Co., Ltd., Shenzhen, China

**Keywords:** Rheumatoid arthritis, Diagnostic imaging, Ultrasonography, Multimodal imaging

## Abstract

**Objectives:**

We aimed to assess the clinical value of multimodal photoacoustic/ultrasound (PA/US) articular imaging scores, a novel imaging method which can reflect the micro-vessels and oxygenation level of inflamed joints of rheumatoid arthritis (RA).

**Methods:**

Seven small joints were examined by the PA/US imaging system. A 0–3 scoring system was used to semi-quantify the PA and power-Doppler (PD) signals, and the sums of PA and PD scores (PA-sum and PD-sum scores) of the seven joints were calculated. The relative oxygen saturation (SO_2_) values of the inflamed joints were measured and classified into 3 PA+SO_2_ patterns. The correlations between the PA/US imaging scores and the disease activity scores were assessed.

**Results:**

Thirty-one patients of RA and a total of 217 joints were examined using the PA/US system. The PA-sum had high positive correlations with the standard clinical scores of RA (DAS28 [ESR] *ρ* = 0.754, DAS28 [CRP] *ρ* = 0.796, SDAI *ρ* = 0.836, CDAI *ρ* = 0.837, *p* < 0.001), which were superior to the PD-sum (DAS28 [ESR] *ρ* = 0.651, DAS28 [CRP] *ρ* = 0.676, SDAI *ρ* = 0.716, CDAI *ρ* = 0.709, *p* < 0.001). For the patients with high PA-sum scores, significant differences between hypoxia and hyperoxia were identified in pain visual analog score (*p* = 0.020) and patient’s global assessment (*p* = 0.026). The PA+SO_2_ patterns presented moderate and high correlation with PGA (*ρ* = 0.477, *p* = 0.0077) and VAS pain score (*ρ* = 0.717, *p* < 0.001).

**Conclusion:**

The PA scores have significant correlations with standard clinical scores for RA, and the PA+SO_2_ patterns are also related with clinical scores that reflect pain severity. PA may have clinical potential in evaluating RA.

**Key Points:**

*• Multimodal photoacoustic/ultrasound imaging is a novel method to assess micro-vessels and oxygenation of local lesions.*

*• Significant correlations between multimodal imaging parameters and clinical scores of RA patients were verified.*

*• The multimodal PA/US system can provide objective imaging parameters, including PA scores of micro-vessels and relative SO*_*2*_ *value, as a supplementary to disease activity evaluation.*

**Supplementary Information:**

The online version of this article (10.1007/s00330-020-07353-z) contains supplementary material, which is available to authorized users.

## Introduction

Rheumatoid arthritis (RA) is a chronic, autoimmune disease marked by symmetrical arthritis that usually involves synovial joints and causes further erosive changes and joint deformities [1, 2]. At present, the widely recognized treatment strategy for RA is “treat-to-target,” in which RA patients are treated and assessed strictly in periods of every 1–3 months until complete remission (CR) or low disease activity (LDA) is achieved [3]. Nevertheless, disease relapses occasionally occur in patients tapering or discontinuing disease-modifying anti-rheumatic drugs (DMARDs), even when they have achieved the treatment goals. This suggests that a more sensitive and objective method is needed to detect subclinical synovitis [4].

Noninvasive imaging methods, including ultrasound (US) and magnetic resonance imaging, are commonly used for the evaluation of joint inflammation [5]. As a convenient and bedside-accessible imaging tool, the role of US has been well established in diagnosing and assessing RA with the development of high-frequency gray-scale ultrasonic probes and power-Doppler ultrasound (PDUS) imaging techniques [6, 7]. Researchers also validated that US played a substantial part in optimizing management of RA patients, as an addition to clinical assessment [8]. Notably, the use of US in RA is still questioned by some researchers [9]. Several studies demonstrated that inflamed synovium could not be completely observed by US, and some PDUS-negative patients might have obvious joint inflammation, which might be ascribed to the insensitivity of PDUS in detecting micro-vessels of the inflammatory lesions [10]. Therefore, new approaches that can enhance the diagnostic performance of US in visualizing arthritis are in great demand.

Photoacoustic imaging (PAI), a hot spot of medical imaging, has broad clinical applications in identifying a variety of different diseases, including thyroid nodules [11], breast cancers [12–14], and inflammatory bowel diseases [15]. The principle of PAI is generating thermal expansion of local tissues induced by laser irradiation, which in turn produces ultrasonic waves that can be detected by common ultrasonic probes. PAI combines the merits of optical imaging and US imaging and reflects optical characteristics of local tissue, including hemoglobin and melanin content, with a deep penetration depth [16, 17]. Moreover, it is applicable to integrate the PA modality into a well-established ultrasonic technique to facilitate dual-modality PA/US imaging after image post-processing, because of the acoustic features of PA signals. A dual-modality PA/US system based on a commercial ultrasonic platform and a handheld probe can be a good way to promote the clinical translation of PAI. By detecting the hemoglobin content in local tissue, PAI can visualize micro-vessels in the hyperplastic synovium of RA-involved joints. Oxygenation can be assessed by calculating the signals of oxygenated and deoxygenated hemoglobin in dual-wavelength PAI and is an important functional indicator of inflamed tissues.

The feasibility of PAI in detecting minor inflammatory lesions in RA joints has been validated by previous studies, in which dual-modality PA/US imaging systems were used [18–25]. In the study by Wang et al, patients with synovitis were recruited for PA scanning of their metacarpophalangeal joints (MCPs) with a dual-modality PA/US system, and significant hyperemia and hypoxia were found in the inflamed synovial tissues [22]. According to van den Berg et al, increased PA signals were detected by a portable PA/US imaging tool in 10 RA patients with clinically evident synovitis and had a good correlation with PDUS [23]. However, the sample sizes of the previous studies were relatively small, and a comprehensive comparison between PA/US imaging and clinical evaluation methods of RA has not been conducted. To explore further the clinical applications and value of PAI in RA, more studies focusing on the clinical evaluation and validation of PAI are necessary.

In this study, we performed imaging the joints of RA patients with a multimodal PA/US imaging system that combined a light device and an ultrasonic transducer. We aimed to assess the correlation of multimodal PA/US articular imaging scores and relative oxygen saturation (SO_2_) values of lesions with different disease activity measurements of RA and to evaluate the potential value of multimodality PA/US imaging of RA in clinical practice.

## Materials and methods

This study was designed as a cross-sectional study. All the procedures in this study were approved by the Institutional Review Board of Peking Union Medical College Hospital (PUMCH) (approval number: JS-1923). Written informed consent was received from all recruited patients.

### Patient recruitment

RA patients aged older than 18 years old were recruited from the outpatient clinic of rheumatology at PUMCH from December 2018 to October 2019. The patients were diagnosed with RA according to the 2010 American College of Rheumatology/European League Against Rheumatism (ACR/EULAR) classification criteria. Patients complicated with other types of arthritis, including osteoarthritis (OA), psoriatic arthritis (ReA), and gouty arthritis, were excluded.

### Multimodal PA/US imaging system

**Fig. 1:**
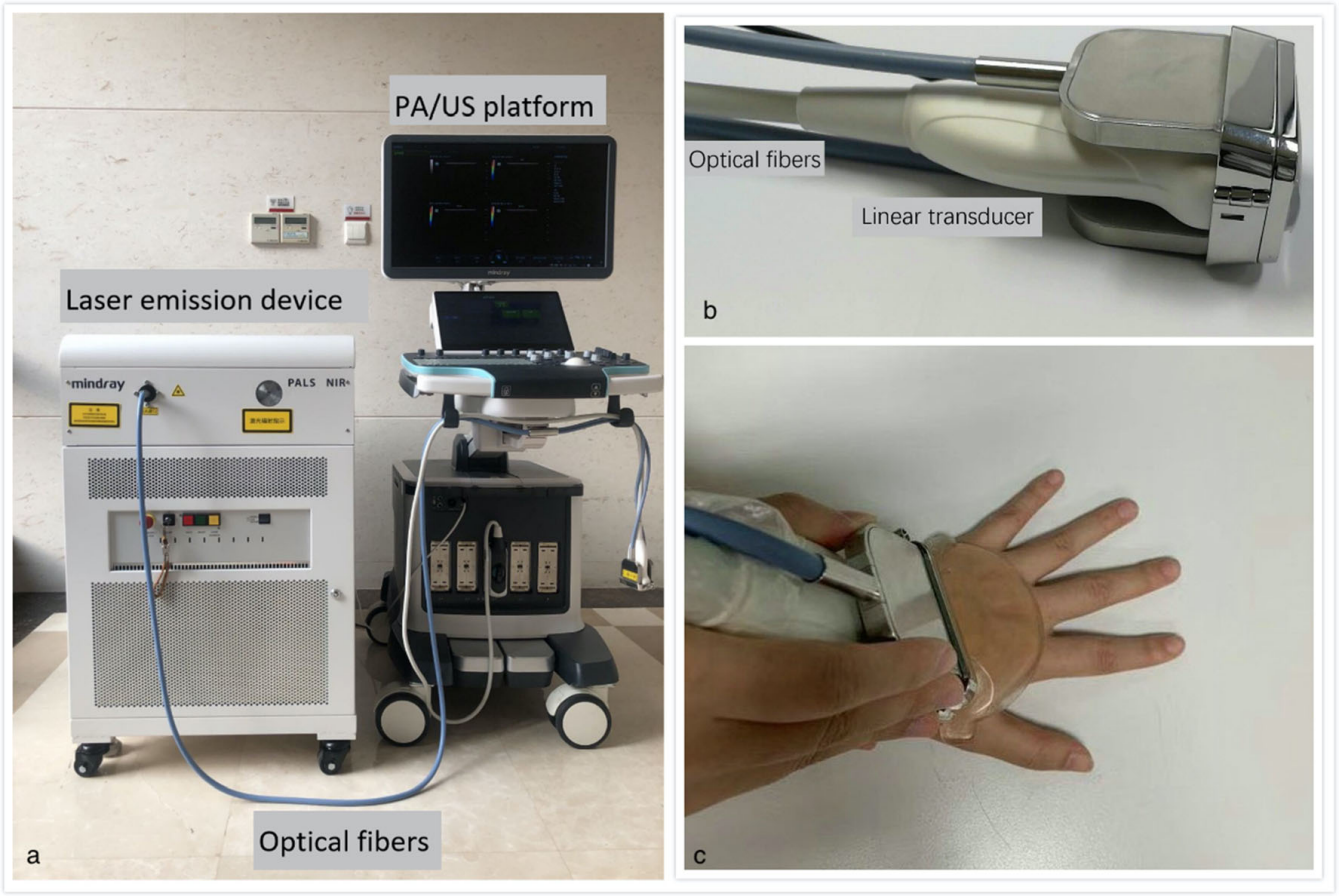
**a** Photograph of PA/US system. A commercial-available ultrasonic equipment (Resona 7, Mindray Bio-Medical Electronics Co., Ltd.) was utilized as the fundamental platform for multimodal imaging, equipped with an OPO tunable laser and handheld linear PA/US probe. **b** Photograph of PA/US probe. A one-two bifurcated fiber bundle was coupled by a custom-made fiber holder to both sides of a linear US transducer with central frequency of 5.8 MHz. **c** Photograph of performing the multimodal PA/US examination using the handheld probe

The schematic of the dual-modality imaging system is presented in Fig. 1a. Commercially available ultrasonic equipment (Resona 7, Mindray Bio-Medical Electronics Co., Ltd.), equipped with an OPO tuneable laser (SpitLight 600-OPO, InnoLas laser GmbH) and a handheld linear probe (L9-3U, Mindray Bio-Medical Electronics Co., Ltd.) (Fig. 1b) centered at 5.8 MHz, was utilized as the fundamental platform of this novel imaging system. Gray-scale US (GSUS) and PDUS could be displayed simultaneously with the PA imaging mode in real time. For PAI, the wavelengths are 750 nm and 830 nm, at which the deoxygenated hemoglobin and oxygenated hemoglobin could reach peak absorption, respectively. Synchronized gray-scale US and 830 nm PAI were provided by the system at a frame of 10 Hz. For power-Doppler US, the following imaging settings were adopted: pulse repetition frequency of 600–1000 Hz, wall filter of 50–100 Hz, maximum gain of 85–90%, scale of 3 cm/s, a rectangle sampling box with no angulation. Detailed information on the imaging system is provided in Supplementary Data S1.

### Imaging protocols

#### Examination procedure

A seven-joint ultrasound scoring system (US7) proposed by Backhaus et al [26] was utilized as the reference to choose joints for the multimodal PA/US examinations. The 2nd metacarpophalangeal joint (MCP2), 3rd metacarpophalangeal joint (MCP3), 2nd proximal interphalangeal joint (PIP2), 3rd proximal interphalangeal joint (PIP3), 2nd metatarsophalangeal joint (MTP2), 5th metatarsophalangeal joint (MTP5), and wrist of the clinically dominant side were chosen for multimodal imaging. GSUS and PDUS imaging of the joints, followed by real-time PA/US imaging, were carried out by a US operator who had 1 year of musculoskeletal US experience and received 1 month of training on operating the system. Detailed explanations of the imaging procedures are presented in Supplementary Data S2, and the training project of the participated radiologists in Supplementary Data S4.

#### Semi-quantitative PDUS and PAI scoring

The semi-quantitative scoring system scored on a scale of 0–3 proposed by Szkudlarek et al [27] for PDUS was utilized as the reference to grade PD and PA images in the study. The global sums of the PDUS (PD-sum, 0–21) and PA (PA-sum, 0–21) scores of the seven joints were calculated for statistical analysis. The images were assessed by two radiologists with 2 years of experience in musculoskeletal US who were blinded to the patients’ information and clinical manifestations of the examined joints. When discrepancies occurred, reconfirmations of the images were conducted until a consensus was reached. After 1 month, the two raters scored the PD and PA images again. The intra-observer of the two times of scoring and inter-observer agreement of the two raters in the first time of scoring were evaluated. The scoring method is explained in detail in Supplementary Data S3.

#### Measurement of SO_2_ and PA+SO_2_ patterns

By calculating the ratio of the pixels of the PA signals in the target areas at wavelengths of 750 nm and 830 nm, the relative SO_2_ values of the inflamed region were determined. The patients were divided into hyperoxic and hypoxic subgroups according to the distribution of the relative SO_2_ values. After calculating the SO_2_ values, the SO_2_ value and the PA-sum score were incorporated as a new index for RA patients, named the “PA+SO_2_ pattern.” The patients with PA-sum scores < 3 were considered to have minimal PA signals, and those with PA scores ≥ 3 were considered to have evident PA signals. According to the PA-sum scores and SO_2_ values, all the patients were divided into 3 groups: pattern 1, no or minimal PA signals; pattern 2, evident PA signals and hyperoxia; and pattern 3, evident PA signals and hypoxia. The associations of the PA+SO_2_ patterns and clinical scores were also evaluated. Detailed explanations of the identification of PA+SO_2_ patterns are presented in Supplementary Data S5.

### Clinical assessment

The relevant clinical information was recorded, including age, sex, disease duration from onset and from diagnosis confirmation, duration of morning stiffness, symptoms, and current medications. The laboratory parameters of the patients were collected, such as the erythrocyte sedimentation rate (ESR), C-reactive protein (CRP), anti-cyclic citrullinated peptide (anti-CCP) antibodies, and rheumatoid factor (RF). For each patient, 28 joints (bilateral PIPs, MCPs, wrists, elbows, shoulders, and knees) were clinically assessed for swelling and tenderness (the swollen joint count (SJC) and the tender joint count (TJC)) by a rheumatologist with 18 years of experience in clinical rheumatology. The visual analog scale (VAS) scores of joint pain and the patient global activity (PGA), evaluator global activity (EGA), disease activity score in 28 joints (DAS28), clinical disease activity index (CDAI), and simplified disease activity index (SDAI) values were also obtained.

### Statistical analysis

The mean ± standard deviation (SD) is used to describe quantitative parameters, including imaging scores, clinical scores, and laboratory data. Correlations between imaging scores (PD-sum scores, PA-sum scores, and the three PA+SO_2_ patterns) and clinical scores were evaluated by Spearman’s rank-order correlation (*ρ*: Spearman’s rank correlation coefficient) [28]. The three PA+SO_2_ patterns (patterns 1, 2, and 3) were regarded as ordinal categorical variables. The correlation coefficient was interpreted as follows: negligible correlation: *ρ* < 0.30; low positive correlation: 0.30 < *ρ* < 0.50; moderate positive correlation: 0.50 < *ρ* < 0.70; high correlation: 0.70 < *ρ* < 0.90; very high positive correlation: *ρ* > 0.90 [29]. The inter-observer and intra-observer agreement of the two radiologists were measured by weighted kappa statistic, presented as *κ* with 95% confidence interval (95% CI). The *κ* value was interpreted as follows: poor: *κ* < 0.20; fair: 0.20 < *κ* < 0.40; moderate: 0.40 < *κ* < 0.60; good: 0.60 < *κ* < 0.80; very good: 0.80 < *κ* < 1.00 [30]. SPSS software (SPSS, 21.0) was used for statistical analysis.

## Results

### Multimodal imaging scores, laboratory data, and clinical scores

A total of 31 patients with RA were recruited for this study, including 24 females and 7 males aged 25–71 years (mean age 51.8, median age 51). A total of 217 joints were examined using the PA/US system. The detailed clinical information of the patients is summarized in Table 1. For the intra-observer agreement of rater 1, *κ* value was 0.87 (0.76–0.96) for two times of PD scoring (*p* = 0.041), and 0.88 (0.79–0.96) for two times of PA scoring (*p* = 0.043), respectively. For the intra-observer agreement of rater 2, *κ* value was 0.92 (0.84–1.00) for PD scoring (*p* = 0.043) and 0.88 (0.81–0.97) for PA scoring (*p* = 0.045), respectively. The intra-observer agreement for both raters in scoring the PD and PA images was very good. And the inter-observer agreement of the two radiologists in scoring the images was also very good (*κ* = 0.83 [0.76–0.93] for PD scoring [*p* = 0.041], and 0.82 [0.71–0.90] for PA scoring [*p* = 0.045]).

**Table 1:** The clinical characteristics, disease activity scores, and imaging scores of the patients of RA

	Mean value ± SD
Age (year)	51.8 ± 12.3
ESR (s)	21.4 ± 26.0
CRP (mg/ml)	12.3 ± 24.5
SJC	7.6 ± 8.0
TJC	7.2 ± 8.1
Pain VAS	24.3 ± 31.2
PGA	26.5 ± 29.0
EGA	22.6 ± 26.6
DAS28 (ESR)	3.9 ± 2.1
DAS28 (CRP)	3.7 ± 2.0
SDAI	21.1 ± 20.1
CDAI	19.6 ± 18.
PD-sum	2.8 ± 3.3
PA-sum	4.5 ± 3.9

*ESR* erythrocyte sedimentation rate, *CRP* C-reactive protein, *DAS28* disease activity score in 28 joints, *SJC* swollen joint count, *TJC* tender joint count, *VAS* visual analog scale, *PGA* patient global activity, *EGA* evaluator global activity, *SDAI* simplified disease activity index, *CDAI* clinical disease activity index, *PD-sum* sum of power-Doppler scores, *PA-sum* sum of photoacoustic scores

**Fig. 2:**
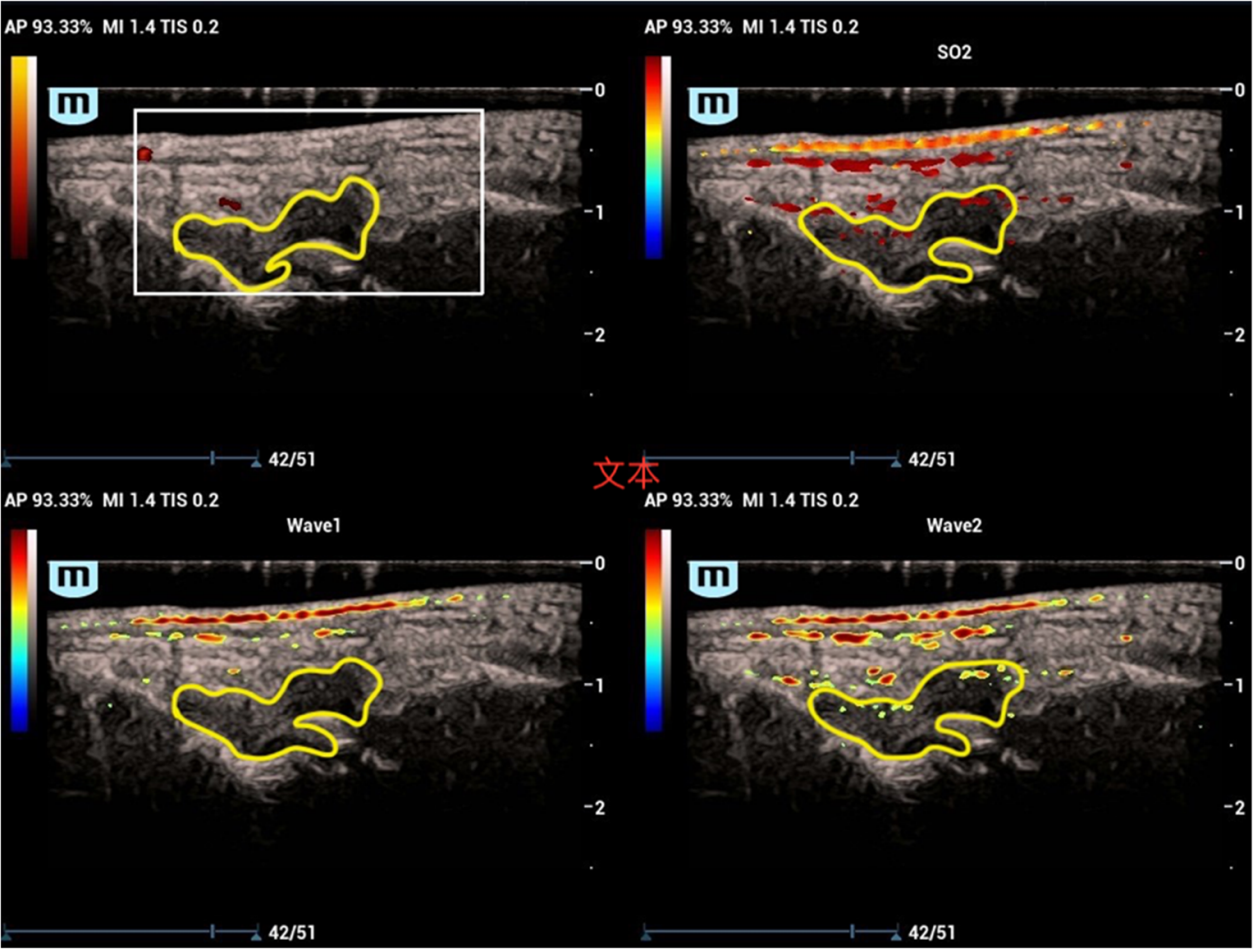
Example of PD 0, PA 1. The PA/US images of the dorsal aspect of the wrist of a 52-year-old female with a 3-year history of RA. PDUS is illustrated in the upper left, PAI of 750 nm and 830 nm in the two inferior parts, and SO_2_ in the upper right. No significant signal was detected in the thickened synovium (hypoechoic region above the bone with a clear boundary, marked with yellow line) of the wrist by PDUS, scored as 0. In the PA images under the wavelength of 830 nm (Wave2), several bars of PA signals inner the hypoechoic region could be visualized. In the SO_2_ part, the signals were demonstrated in the pseudo-color of red, with a relative SO_2_ value of 93.52%, classified as hyperoxia. The PA signals in the skin layer were generated by the optical absorption of melanin

**Fig. 3:**
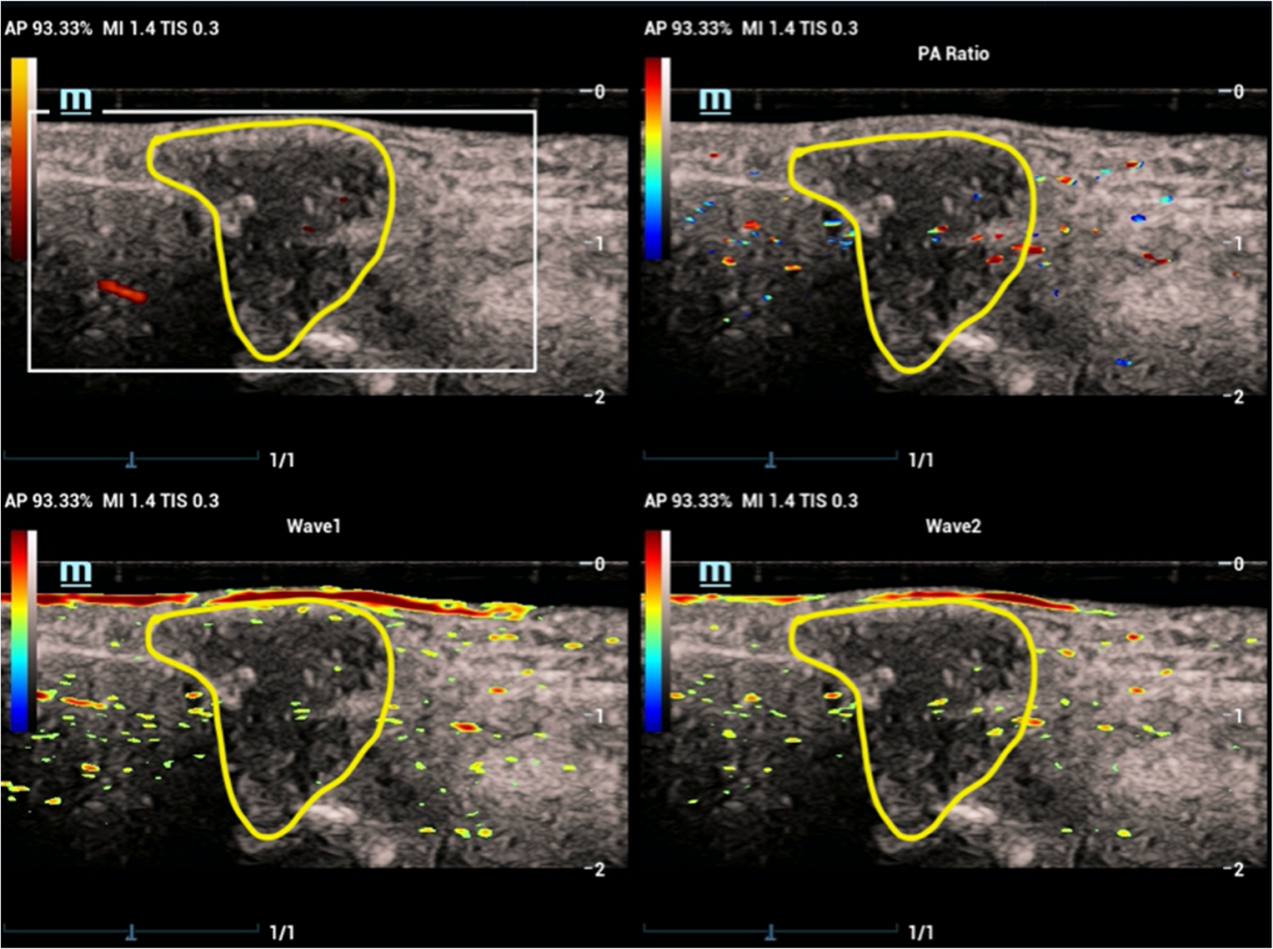
Example of PD 1, PA 2. The PA/US images of the dorsal aspect of the wrist of a 69-year-old male with a 20-year history of RA. The synovium was significantly thickened, presented as hypoxic area above the hyperechoic line of wrist bones (the region marked with the yellow line). Only a few vessels were detected by PDUS in the margin of the lesion, and the PD imaging was scored as grade 1. PA signals were distributed in both the center and the margin of the lesion at both of the wavelengths (Wave1 and Wave2), ranked with a score of 2. The signals in the SO_2_ part were presented as a mixture of red and blue color and calculated with a relative SO_2_ value of 79.30%, classified as hypoxia

**Fig. 4:**
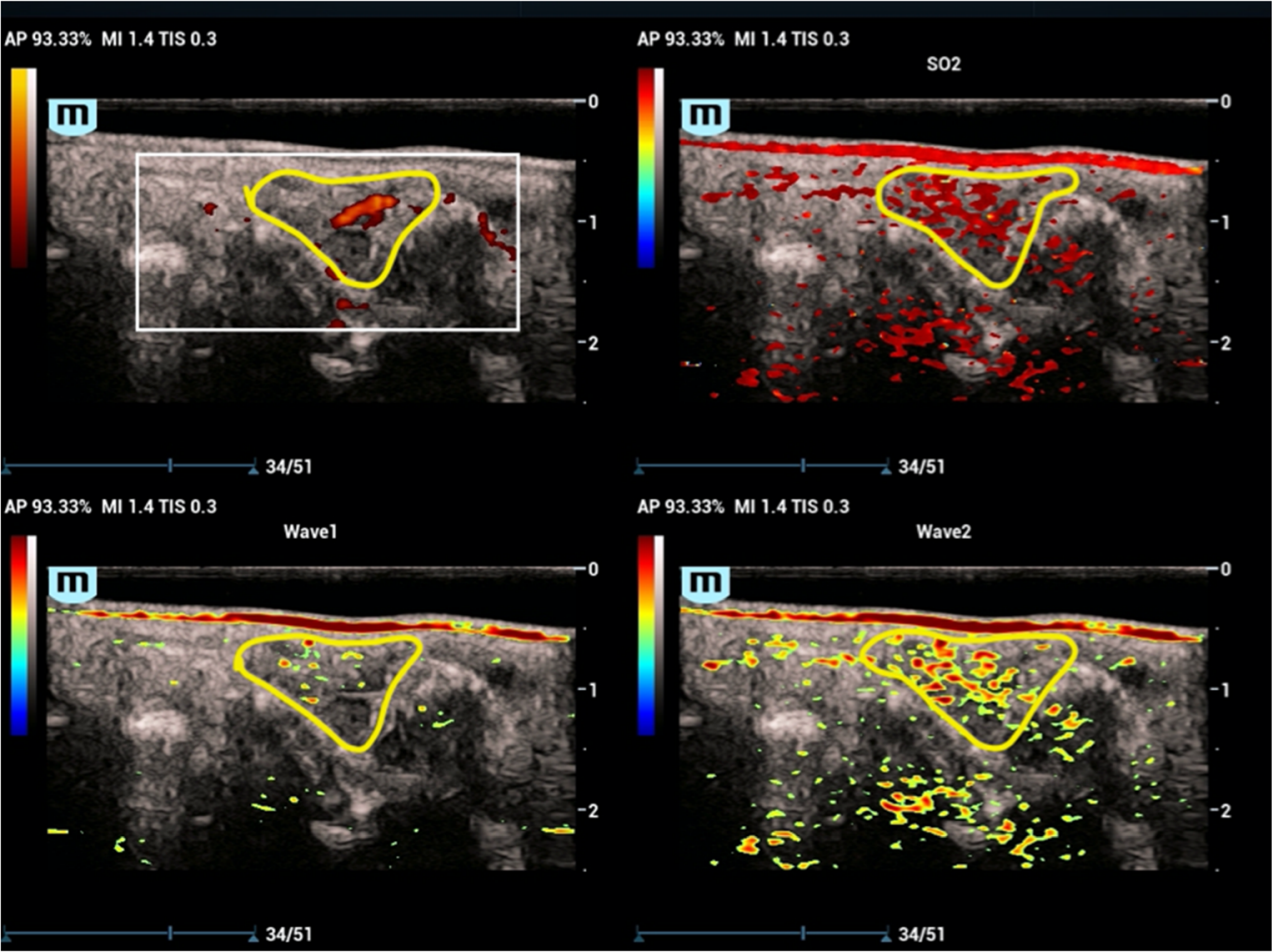
Example of PD 2, PA 3. The PA/US images of the dorsal aspect of the wrist of a 45-year-old female with a 3-year history of RA. Significant signals were presented in the PD mode, scored as 2. In the PA mode, abundant optical signals were visualized under both of the two wavelengths, representing hyperemia of the inflamed lesion (the region marked with the yellow line). We scored the PA results of the wrist as 1. In the SO_2_ part, the signals were demonstrated in the pseudo-color of red, with a relative SO_2_ value of 97.33%, classified as hyperoxia

A total of 217 joints were examined using the PA/US system, and the numbers of each PA grading of the corresponding PD grading (0–3) are shown in Table 2. Among all those small joints, a total of 16 joints were divided into the highest level of both PA and PD. There were 21 joints scored as grade 1 in PA, showing no signals in PD. The imaging interpretation of the PD and PA results is shown in Figs. 2, 3, and 4.

**Table 2:** The numbers of small joints according to the 1–3 PA score and the corresponding PD score

PA score	PD score				Total
	0	1	2	3	
0	149	0	0	0	149
1	21	4	0	0	25
2	7	4	10	0	21
3	0	2	4	16	22
Total	177	10	14	16	217

*PA* photoacoustic, *PD* power-Doppler

### The correlation between PA/PD scores and RA disease activity measurements

**Fig. 5:**
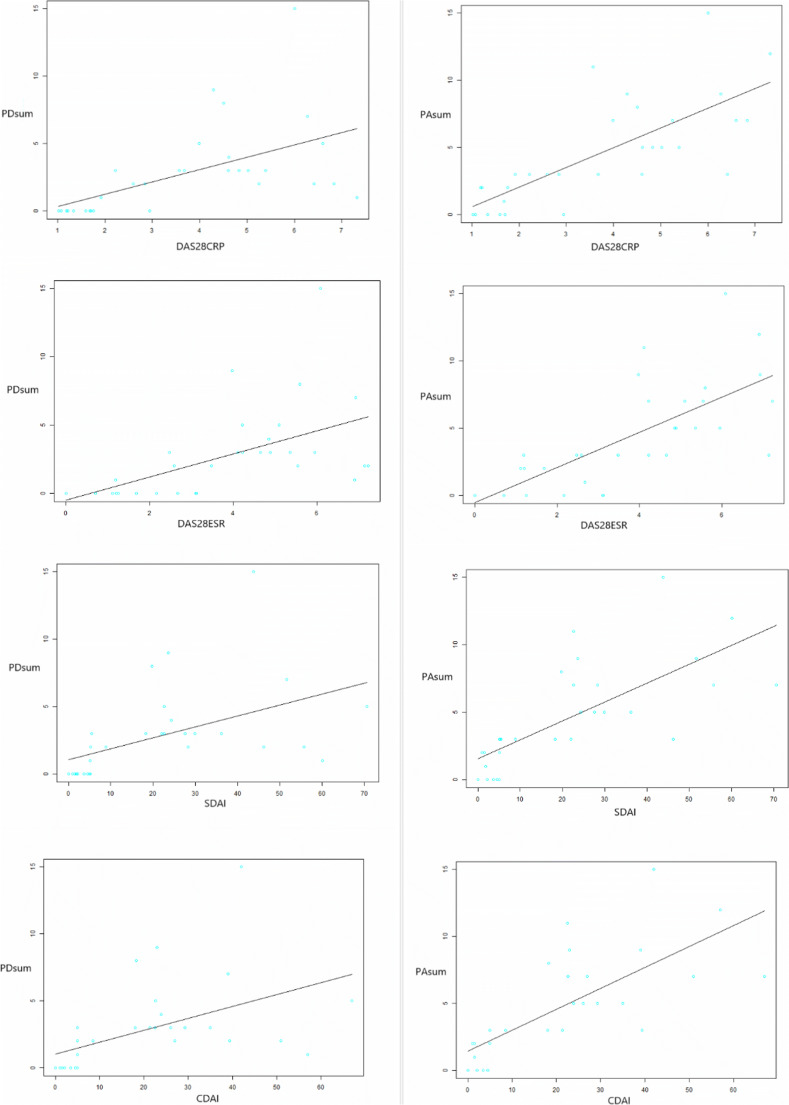
The fitting curves and scatter plots of imaging scores (PD-sum and PA-sum, Y-axis) and clinical scores (DAS28ESR, DAS28CRP, CDAI, SDAI for the X-axis, respectively). An upward straight line reflecting the correlation between the imaging parameter and the clinical score was observed in each figure, respectively, indicating significant correlations of PA scores and standard clinical scores (DAS28, SDAI, CDAI)

The correlations among the PA-sum scores, PD-sum scores, and RA disease activity measurements are listed in Table 3. The PA-sum had high positive correlations with the DAS28 (ESR) (*ρ* = 0.754 [0.546–0.875], *p* < 0.0001), DAS28 (CRP) (*ρ* = 0.796 [0.615–0.897], *p* < 0.0001), SDAI (*ρ* = 0.836 [0.684–0.918], *p <* 0.0001), and CDAI (*ρ* = 0.837 [0.689–0.919], *p* < 0.0001), which were all superior to the PD-sum (DAS28 [ESR] *ρ* = 0.651 [0.385–0.817], *p* = 0.0001; DAS28 [CRP] *ρ* = 0.676 [0.422–0.831], *p* < 0.0001; SDAI *ρ* = 0.716 [0.484–0.854], *p* < 0.0001; and CDAI *ρ* = 0.709 [0.573–0.850], *p* < 0.0001). The PA-sum presented high positive correlations with TJC and SJC (*ρ* = 0.801 [0.620–0.901], *p* < 0.0001; *ρ* = 0.792 [0.604–0.896], *p* < 0.0001), respectively). The PD-sum had moderate to high correlations with TJC and SJC (*ρ* = 0.719 [0.484–0.857], *p* < 0.0001; *ρ* = 0.699 [0.453–0.846], *p* < 0.0001, respectively). The PA-sum had moderate positive correlation with CRP (*ρ* = 0.544 [0.235–0.753], *p* = 0.0016); and the PD-sum had low positive correlation with CRP (*ρ* = 0.432 [0.0922–0.682], *p* = 0.0151). Neither the PD-sum nor the PA-sum correlated with ESR. The PA-sum scores had moderate correlation with the patient’s VAS pain score (*ρ* = 0.698 [0.451–0.846], *p* < 0.0001), of which the correlation coefficient was higher than that of the PD-sum (*ρ* = 0.508 [0.181–0.734], *p* = 0.0041). The PA-sum had low correlation with global assessment (PGA, *ρ* = 0.482 [0.147–0.718], *p* = 0.0070), while the PD-sum scores were not correlated with PGA. The fitted curves of the PD/PA scoring results and clinical scores are shown in Fig. 5. An upward trend could be observed in each curve, which validated the correlations between the imaging and clinical results.

**Table 3:** The correlation between PD/PA-sum scores and RA disease activity measurements

		Correlation	95% CI	*p* value
ESR	PD-sum	0.211	− 0.154–0.526	0.254
	PA-sum	0.264	− 0.0992–0.566	0.262
CRP	PD-sum	0.432	0.0922–0.682	0.0151
	PA-sum	0.544	0.235–0.753	0.0016
SJC	PD-sum	0.699	0.453–0.846	< 0.0001
	PA-sum	0.792	0.604–0.896	< 0.0001
TJC	PD-sum	0.719	0.484–0.857	< 0.0001
	PA-sum	0.801	0.620–0.901	< 0.0001
Pain VAS	PD-sum	0.508	0.181–0.734	0.0041
	PA-sum	0.698	0.451–0.846	< 0.0001
PGA	PD-sum	0.196	− 0.176–0.520	0.298
	PA-sum	0.482	0.147–0.718	0.0070
EGA	PD-sum	0.421	0.0712–0.678	0.0206
	PA-sum	0.622	0.338–0.803	0.0022
DAS28 (ESR)	PD-sum	0.651	0.385–0.817	0.0001
	PA-sum	0.754	0.546–0.875	< 0.0001
DAS28 (CRP)	PD-sum	0.676	0.422–0.831	< 0.0001
	PA-sum	0.796	0.615–0.897	< 0.0001
SDAI	PD-sum	0.716	0.484–0.854	< 0.0001
	PA-sum	0.836	0.684–0.918	< 0.0001
CDAI	PD-sum	0.709	0.473–0.850	< 0.0001
	PA-sum	0.837	0.689–0.919	< 0.0001

*ESR* erythrocyte sedimentation rate, *CRP* C-reactive protein, *DAS28* disease activity score in 28 joints, *SJC* swollen joint count, *TJC* tender joint count, *VAS* visual analog scale, *PGA* patient global activity, *EGA* evaluator global activity, *SDAI* simplified disease activity index, *CDAI* clinical disease activity index, *PD-sum* sum of power-Doppler scores, *PA-sum* sum of photoacoustic scores, *95% CI* 95% confidence interval

### PA+SO_2_ patterns and their correlations with RA disease activity measurements

**Fig. 6:**
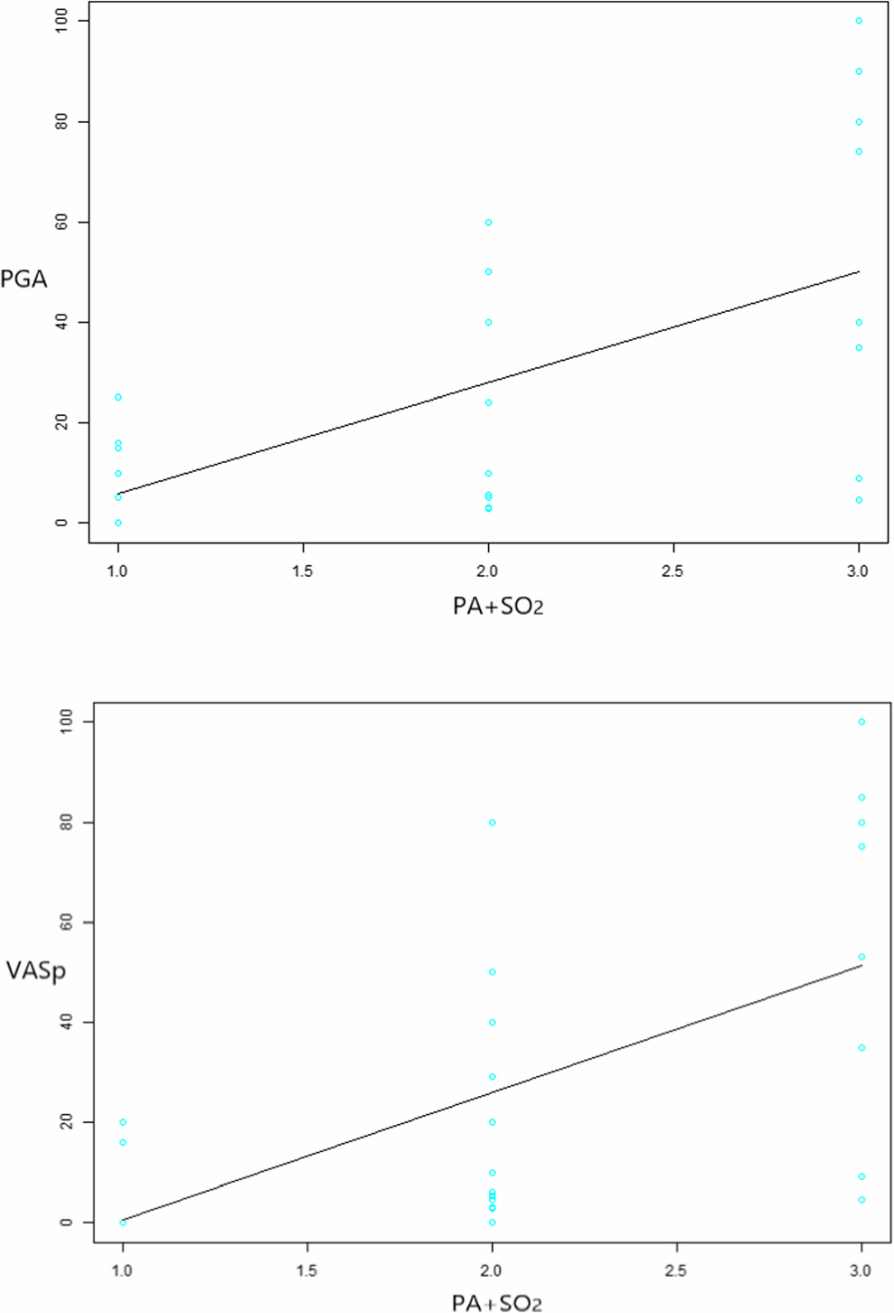
The fitting curves and scatter plots of the clinical scores (pain VAS and PGA, Y-axis) and the three PA+SO_2_ patterns (X-axis). Highest degree of pain VAS and PGA presented in pattern 3. Significant correlations between the clinical scores (pain VAS and PGA) of the three PA+SO_2_ patterns could be identified

Among the 31 patients, relative SO_2_ values were calculated for a total of 21 patients with evident PA signals. The 10 cases who had no detectable PA signals within inflamed tissues with a PA-sum of 0 were excluded for measurement of SO_2_. The SO_2_ value of the small joints was 87.5 ± 10.1%. The patients were divided into the hyperoxic subgroup and the hypoxic subgroup according to the distribution of the relative SO_2_ values. Twelve patients were classified as hyperoxia with the relative SO_2_ value greater than 90%, and nine patients were hypoxia with the value smaller than 85% (Supplementary Data S4 and Fig. S1). And 10 patients with no or minimal PA signals were classified as pattern 1, 12 patients with evident PA signals and hyperoxia classified as pattern 2, and 9 patients with evident PA signals and hypoxia classified as pattern 3. The clinical scores of the three patterns are shown in Table 4. Significant differences in VAS pain score (*p* = 0.020) and PGA (*p* = 0.026) were detected between patients with evident PA signals and those who were classified as hypoxic and hyperoxic (patterns 2 and 3). No differences could be identified in the other indexes among patients. The PA+SO_2_ patterns presented high positive correlation with VAS pain score (*ρ* = 0.717 [0.482–0.856], *p* < 0.0001) and moderate correlation with PGA (*ρ* = 0.477 [0.141–0.714], *p* = 0.0077) (Fig. 6).

**Table 4:** The clinical scores of the PA+SO_2_ patterns

PA+SO_2_					
	Pattern 1 (*n* = 10)	Pattern 2 (*n* = 12)	Pattern 3 (*n* = 9)	*p* value*	*p* value**
ESR	12.9 ± 13.5	25.7 ± 31.1	25.1 ± 29.5	0.2207	0.507
CRP	2.3 ± 4.7	16.1 ± 35.1	18.4 ± 18.3	< 0.001	0.858
SJC	0.6 ± 1.0	10.8 ± 7.7	11.4 ± 8.3	< 0.001	0.883
TJC	0.4 ± 1.0	10.1 ± 7.3	11.5 ± 9.1	< 0.001	0.704
Pain VAS	3.6 ± 7.6	20.9 ± 24.7	55.2 ± 35.9	< 0.001	0.020
PGA	9.1 ± 8.2	22.6 ± 21.4	54.1 ± 36.9	0.0077	0.026
EGA	5.5 ± 3.7	24.1 ± 27.5	41.6 ± 30.0	0.0046	0.195
DAS28 (ESR)	1.7 ± 1.0	4.5 ± 1.6	5.3 ± 1.5	< 0.001	0.256
DAS28 (CRP)	1.5 ± 0.6	4.2 ± 1.4	5.4 ± 1.5	< 0.001	0.091
SDAI	2.5 ± 1.9	23.6 ± 14.8	38.3 ± 20.7	< 0.001	0.074
CDAI	2.3 ± 1.8	21.9 ± 12.7	35.9 ± 19.1	< 0.001	0.058

*p value of clinical parameters among the three PA+SO_2_ patterns

**p value of the difference in clinical parameters between pattern 2 and pattern 3

*ESR* erythrocyte sedimentation rate, *CRP* C-reactive protein, *DAS28* disease activity score in 28 joints, *SJC* swollen joint count, *TJC* tender joint count, *VAS* visual analog scale, *PGA* patient global activity, *EGA* evaluator global activity, *SDAI* simplified disease activity index, *CDAI* clinical disease activity index, *SO*_*2*_ oxygen saturation

## Discussion

In this study, a multimodal PA/US imaging system was utilized to evaluate the small joints of RA patients with different disease activities. The PA parameters, including PA-sum scores and PA+SO_2_ patterns, correlated well with the clinical scores, suggesting the feasibility of PAI in assessing the disease activity of RA. In addition to conventional gray-scale US and PDUS, PAI may be utilized as a new modality affiliated with US systems to provide new imaging information to assist in assessing the disease activity of RA.

We performed an overall imaging evaluation of small joints in patients with RA, including the MCP, PIP, MTP, and wrist, using a 0–3 PDUS grading scale and the simplified US7 scoring system as a reference [26, 31]. The PA-sum scores of the seven joints were significantly higher than the PD-sum scores, indicating that PAI was more sensitive to small vessels in the hypertrophied synovium and inflamed tendon sheath than PDI. For lesions with a PD score of 3, the highest grade, abundant PA signals could also be seen. For the hypertrophic regions of the synovium with a low PD score, including 0 and 1, evident PA signals could be found in some joints, implying that, in some RA cases, active inflammation that could be detected by PAI may not be detected by conventional US examinations. The PD scores were indicated by this study to have a moderate to high correlation with the clinical scores, with a correlation coefficient of 0.49–0.71. This result was consistent with the coefficient of 0.46–0.63 in previous studies [32–36]. Compared with the PD-sum score, the PA-sum score presented higher correlation coefficient, which had high positive correlation with the standard clinical disease activity scores (*ρ* > 0.70 for DAS28, SDAI, and CDAI). Therefore, PAI may be able to accurately reflect the disease activity of the individual patients, which is especially useful for active lesions that are difficult to recognize by PDUS.

Hypoxia has been believed to be a hallmark of inflammatory diseases in this decade, and RA is no exception [37]. In vivo partial pressure of oxygen (pO_2_) measurements of RA patients have also been performed using arthroscopy and ion electrodes by Ursula Fearo et al, but the method was relatively time-consuming and invasive [38]. In this study, we classified the patients into hyperoxia and hypoxia subtypes using the relative SO_2_ values, identified from pixels of PA signals. We found that, among the joints with higher levels of PA signals, the hypoxic individuals were more likely to have higher VAS and PGA scores than the hyperoxic patients. A high positive correlation was validated between the combined PA+SO_2_ patterns and pain VAS, and moderate correlation of PA+SO_2_ patterns and PGA scores, indicating that the oxygenation status might be related with the pain severity of the joints. The relative SO_2_ value could be used as a supplementary to assessing joint symptoms. No significant differences were detected between the PA+SO_2_ pattern and the other indexes. This result was not strong enough to establish the role of oxygenation in evaluating disease activity, which might be caused by the limited cases enrolled in this preliminary study. Studies about measuring oxygenation using PAI with larger sample size are required for further verifying the value of dual-wavelength PA in evaluating RA.

The multimodal PA/US system developed by our team integrated the PA modality into a high-end commercial US unit, making it possible to display real-time multimodal imaging and provide versatile imaging information for target lesions. A handheld probe combining the emission of lasers and ultrasonic waves and reception of US and PA signals was also equipped with the system, making the system easy for radiologists to operate. In addition, the PA and SO_2_ scoring system in our study was created using built-in software, with reference to the widely approved 0–3 PD scores and US7 scoring systems, thus providing a concise and systematic evaluation of RA patients that could be more convenient and acceptable for clinicians.

There still exist several limitations in our study. First, the sample of RA patients was still small, and more patients must be recruited for further validation. Further clinical studies with larger sample sizes are also expected to explore the value of SO_2_ in the diagnosis of RA. Second, this study is a preliminary cross-sectional observational study of the multimodal PA/US imaging system, and whether these PA measures can predict treatment response and risk of RA relapse remains to be explored by a prospective cohort study in the future.

## Conclusions

The correlations between PA scores of micro-vessels and standard clinical scores for RA were identified, and relative SO_2_ was also related with clinical scores that reflect pain severity. The multimodal PA/US imaging system provided comprehensive imaging parameters and might have great potential in the evaluation of disease activity of RA patients.

## Supplementary Information

ESM 1 (DOCX 51 kb)

## Abbreviations

ACR/EULAR: American College of Rheumatology/European League Against Rheumatism
anti-CCP: Anti-cyclic citrullinated peptide antibodies
CDAI: Clinical disease activity index
CDUS: Color-Doppler US imaging
CR: Complete remission
CRP: C-reactive protein
DAS28: Disease activity score in 28 joints
DMARDs: Disease-modifying anti-rheumatic drugs
EGA: Evaluator’s global assessment
ESR: Erythrocyte sedimentation rate
GSUS: Gray-scale US
HIFs: Hypoxia-induced factors
LDA: Low disease activity
MCP: Metacarpophalangeal
MTP: Metatarsophalangeal
OA: Osteoarthritis
PA: Photoacoustic
PAI: Photoacoustic imaging
PD: Power-Doppler
PDUS: Power-Doppler ultrasound
PGA: Patient’s global assessment
PIP: Proximal interphalangeal
RA: Rheumatoid arthritis
ReA: Psoriatic arthritis
RF: Rheumatoid factor
SD: Standard deviation
SDAI: Simplified disease activity index
SJC: Swelling joint count
SO_2_: Oxygen saturation
TJC: Tender joint count
US: Ultrasound
US7: Seven-joint ultrasound scoring system
VAS: Visual analog score
95%CI: 95% confidence interval

## References

[1] Scott DL, Wolfe F, Huizinga TW (2010). Rheumatoid arthritis. Lancet.

[2] Smolen JS, Aletaha D, McInnes IB (2016). Rheumatoid arthritis. Lancet.

[3] Smolen JS, Breedveld FC, Burmester GR (2016). Treating rheumatoid arthritis to target: 2014 update of the recommendations of an international task force. Ann Rheum Dis.

[4] Kuijper TM, Luime JJ, de Jong PH (2016). Tapering conventional synthetic DMARDs in patients with early arthritis in sustained remission: 2-year follow-up of the tREACH trial. Ann Rheum Dis.

[5] Colebatch AN, Edwards CJ, Ostergaard M (2013). EULAR recommendations for the use of imaging of the joints in the clinical management of rheumatoid arthritis. Ann Rheum Dis.

[6] Forien M, Ottaviani S (2017). Ultrasound and follow-up of rheumatoid arthritis. Joint Bone Spine.

[7] Sakellariou G, Conaghan PG, Zhang W (2017). EULAR recommendations for the use of imaging in the clinical management of peripheral joint osteoarthritis. Ann Rheum Dis.

[8] Ciurtin C, Jones A, Brown G (2019). Real benefits of ultrasound evaluation of hand and foot synovitis for better characterisation of the disease activity in rheumatoid arthritis. Eur Radiol.

[9] Caporali R, Smolen JS (2018). Back to the future: forget ultrasound and focus on clinical assessment in rheumatoid arthritis management. Ann Rheum Dis.

[10] Nguyen H, Ruyssen-Witrand A, Gandjbakhch F, Constantin A, Foltz V, Cantagrel A (2014). Prevalence of ultrasound-detected residual synovitis and risk of relapse and structural progression in rheumatoid arthritis patients in clinical remission: a systematic review and meta-analysis. Rheumatology (Oxford).

[11] Yang M, Zhao L, He X (2017). Photoacoustic/ultrasound dual imaging of human thyroid cancers: an initial clinical study. Biomed Opt Express.

[12] Neuschler EI, Butler R, Young CA et al (2017) A pivotal study of optoacoustic imaging to diagnose benign and malignant breast masses: a new evaluation tool for radiologists. Radiology. 10.1148/radiol.2017172228:172228 10.1148/radiol.2017172228 29178816

[13] Asao Y, Hashizume Y, Suita T (2016). Photoacoustic mammography capable of simultaneously acquiring photoacoustic and ultrasound images. J Biomed Opt.

[14] Di Leo G, Trimboli RM, Sella T, Sardanelli F (2017). Optical imaging of the breast: basic principles and clinical applications. AJR Am J Roentgenol.

[15] Knieling F, Neufert C, Hartmann A (2017). Multispectral optoacoustic tomography for assessment of Crohn’s disease activity. N Engl J Med.

[16] Wang LV, Hu S (2012). Photoacoustic tomography: in vivo imaging from organelles to organs. Science.

[17] Li C, Wang LV (2009). Photoacoustic tomography and sensing in biomedicine. Phys Med Biol.

[18] Wang X, Chamberland DL, Carson PL (2006). Imaging of joints with laser-based photoacoustic tomography: an animal study. Med Phys.

[19] Rajian JR, Shao X, Chamberland DL, Wang X (2013). Characterization and treatment monitoring of inflammatory arthritis by photoacoustic imaging: a study on adjuvant-induced arthritis rat model. Biomed Opt Express.

[20] Xu G, Rajian JR, Girish G (2013). Photoacoustic and ultrasound dual-modality imaging of human peripheral joints. J Biomed Opt.

[21] Beziere N, von Schacky C, Kosanke Y (2014). Optoacoustic imaging and staging of inflammation in a murine model of arthritis. Arthritis Rheumatol.

[22] Jo J, Xu G, Cao M (2017). A functional study of human inflammatory arthritis using photoacoustic imaging. Sci Rep.

[23] van den Berg PJ, Daoudi K, Bernelot Moens HJ, Steenbergen W (2017). Feasibility of photoacoustic/ultrasound imaging of synovitis in finger joints using a point-of-care system. Photoacoustics.

[24] Zhu Y, Xu G, Yuan J (2018). Light emitting diodes based photoacoustic imaging and potential clinical applications. Sci Rep.

[25] Jo J, Tian C, Xu G (2018). Photoacoustic tomography for human musculoskeletal imaging and inflammatory arthritis detection. Photoacoustics.

[26] Backhaus M, Ohrndorf S, Kellner H (2009). Evaluation of a novel 7-joint ultrasound score in daily rheumatologic practice: a pilot project. Arthritis Rheum.

[27] Szkudlarek M, Narvestad E, Klarlund M, Court-Payen M, Thomsen HS, Ostergaard M (2004). Ultrasonography of the metatarsophalangeal joints in rheumatoid arthritis: comparison with magnetic resonance imaging, conventional radiography, and clinical examination. Arthritis Rheum.

[28] McPherson K (2005). 1. Statistical methods for rates and proportions (3rd edn). Joseph L. Fleiss, Bruce Levin and Myunghee Cho Paik, Wiley, New Jersey, 2003. No. of pages: xxvii + 760. Price: $99.95 (hardcover). ISBN: 0-471-52629-0. Stat Med.

[29] Hinkle DE, Wiersma W, Jurs SG (2003) Applied statistics for the behavioral sciences. Houghton Mifflin College Division

[30] Ashby D (1991). Practical statistics for medical research. Douglas G. Altman, Chapman and Hall, London, 1991. No. of pages: 611. Price: £32.00. Stat Med.

[31] Backhaus TM, Ohrndorf S, Kellner H (2013). The US7 score is sensitive to change in a large cohort of patients with rheumatoid arthritis over 12 months of therapy. Ann Rheum Dis.

[32] Vlad V, Berghea F, Libianu S (2011). Ultrasound in rheumatoid arthritis: volar versus dorsal synovitis evaluation and scoring. BMC Musculoskelet Disord.

[33] Yamada Y, Ogasawara M, Gorai M (2016). The synovial grade corresponding to clinically involved joints and a feasible ultrasound-adjusted simple disease activity index for monitoring rheumatoid arthritis. Mod Rheumatol.

[34] Yokota K, Tsuzuki Wada T, Akiyama Y, Mimura T (2018). Detection of synovial inflammation in rheumatic diseases using superb microvascular imaging: Comparison with conventional power Doppler imaging. Mod Rheumatol.

[35] Zufferey P, Moller B, Brulhart L (2014). Persistence of ultrasound synovitis in patients with rheumatoid arthritis fulfilling the DAS28 and/or the new ACR/EULAR RA remission definitions: results of an observational cohort study. Joint Bone Spine.

[36] Naredo E, Bonilla G, Gamero F, Uson J, Carmona L, Laffon A (2005). Assessment of inflammatory activity in rheumatoid arthritis: a comparative study of clinical evaluation with grey scale and power Doppler ultrasonography. Ann Rheum Dis.

[37] Ng CT, Biniecka M, Kennedy A (2010). Synovial tissue hypoxia and inflammation in vivo. Ann Rheum Dis.

[38] Fearon U, Canavan M, Biniecka M, Veale DJ (2016). Hypoxia, mitochondrial dysfunction and synovial invasiveness in rheumatoid arthritis. Nat Rev Rheumatol.

