# Supplementary material for: Multimodal photoacoustic/ultrasonic imaging system: a promising imaging method for the evaluation of disease activity in rheumatoid arthritis
Source: Eur Radiol. 2020 Nov 12;31(5):3542–52. doi: 10.1007/s00330-020-07353-z (PMC8043900; doi:10.1007/s00330-020-07353-z)
Supplement: Supplementary file 1 — (DOCX 51 kb) [file 330_2020_7353_MOESM1_ESM.docx]

**Supplementary Data S1. Detailed information about the multi-modality PA/US imaging system**

A commercial-available ultrasonic equipment (Resona 7, Mindray Bio-Medical Electronics Co., Ltd.) was utilized as the fundamental platform of this novel imaging system. A variety of imaging modalities, including grey-scale US (GSUS), color-Doppler US imaging (CDUS) and power-Doppler US imaging (PDUS), were equipped in this system, and could be displayed simultaneously with the PA imaging mode. A 192-element handheld probe with a central frequency of 5.8 MHz was used for emitting and receiving ultrasonic beams (L9-3U, Mindray Bio-Medical Electronics Co., Ltd.) (Supplementary Figures S1). A one-two bifurcated optical fiber bundle (Ceramoptec GmbH), which was mounted by a custom-made holder onto the both sides of the probe, was used to deliver the laser. The laser was emitted to tissues while the generated photoacoustic signals, which presented as the form of ultrasonic waves, were detected by the linear ultrasonic transducer. An OPO tunable laser (Spitlight 600-OPO, Innolas laser GmbH), which generated 680-980 nm laser pulses at 10Hz, were utilized. In this study, 750 nm and 830 nm wavelengths were selected for PA imaging, at which the deoxygenated hemoglobin and oxygenated hemoglobin could reach the peak absorption respectively. The imaging system could provide combined PA/US imaging at 10 Hz frame rate for single wavelength, and 5 Hz for dual wavelengths. Real-time oxygen saturation (SO_2_) mapping with a frame rate of 5 Hz was also provided. In the PA/US dual modality imaging mode, the US and PA modules ran in a time division multiplexing approach, the PA module was synchronized with the laser pulses, and the US module operated in the periods between the successive laser pulses.

The real-time imaging area on the screen was divided into 4 parts for the multimodal imaging. The upper left part showed the conventional US image, including GSUS, CDUS or PDUS. The two bottom images were PA images superimposed on grey-scale US images, at the wavelength of 750nm and 830nm, respectively. The upper right part was the mapping of SO_2_ in the form of pseudo-color, which reflected the oxygenation by integrating the signals from the two PA images at the wavelengths of 750nm and 830nm. In SO_2_ image, the red signals represented hyperoxia, and the blue signals represented hypoxia of the local tissues. To better discriminate the oxygenation situation of different cases, the pixels of PA signals at the wavelengths of 750nm and 830nm were measured to generate quantified values for representing SO_2_ level of the targeted regions.

Before formal experiments, multimodal imaging was performed on healthy volunteers for establishing the most suitable imaging settings for PA imaging. For PA imaging, signals behind bone surfaces was deemed as noises originated from the light reflection of bones, which would be excluded from imaging interpretation. Imaging gain of 45-55 was set for minimize the PA noises, using the pseudo-signals behind bone surfaces as the reference. White protective shell and white plane for examination were also utilized for reducing noises and acquiring better PA imaging. The strong linear PA signals beneath the skin was also figured out and excluded during imaging scoring.

**Supplementary Data S2. Imaging procedures**

The 2^nd^ metacarpophalangeal joint (MCP2), the 3^rd^ metacarpophalangeal joint (MCP3), the 2^nd^ proximal interphalangeal joint (PIP2), the 3^rd^ proximal interphalangeal joint (PIP3), the 2^nd^ metatarsophalangeal joint (MTP2), the 5^th^ metatarsophalangeal joint (MTP5), and wrist of the clinically dominant side were chosen for the multimodal imaging. The imaging examination was performed at a temperature of 20-25℃ and a humidity of 50-70%. Laser safety glasses have been prepared for the operator and the patients before examination. During the process of imaging examination, the patients sit beside the operator and put their hands on a white plane, which was placed on an examining table beside the imaging system. The probe was placed on the dorsal side of the fingers, toes and wrists in the long axis, with a gel pat in between (Supplementary Figure S1). Detailly, the dorsal aspect of MCP, PIP and MTP joints were scanned in the longitudinal plane. As for the wrist joints, the radiocarpal joints, intercarpal joints and the distal ulna were all longitudinally scanned over the dorsal aspect. Firstly, GSUS and PDUS scanning of the joints were carried out by a US operator, who had one-year of musculoskeletal US experience and received one-month training for system operating. Afterwards, a real-time PA/US imaging was implemented for each joint by the same operator. The depth for visualizing MCP, MTP and PIP joints was set at 2 cm, and the depth for wrist was 2.5-3 cm. During the PA scanning, the first part of the screen was be switched to different US modes, to make a complemented comparison of US and PA imaging. The examining time of conventional US was about 30 seconds to 1 minute, and the time for multimodal PA/US imaging was about 2 minutes for each joint. The examining time for the whole imaging process was approximately 20-30 minutes. The imaging settings for US and PA were kept unchanged throughout the inspection. At least five PA/US images in the long axis with discernable lesions for those inflamed joints and normal structures for the uninfected joints were recorded for further reviewing.

**Supplementary Data S3. The PD/PA scoring method**

The 0-3 scoring system was utilized: Score 0, no PD/PA signals; Score 1, minimal (less than 3 bars) PD/PA signals in the inflamed regions (the area of low echogenicity in the hypertrophied synovium, tenosynovitis, paratenonitis); Score 2, PD/PA signals in less than half of the inflamed regions; Score 3, PD/PA signals in more than half of the inflamed regions. Firstly, the inflammatory lesions, including the thickened synovium, the hypertrophic tendons and the abnormal hypoechoic areas besides the tendons or within the tendon sheaths, were extracted and identified as synovitis and tenosynovitis/paratenonitis in the examined joints by GSUS. Then the PDUS and PA imaging of the inflammatory areas was semi-quantitatively scored according to the scoring system for each joint. The images in the first part of the screen in PDUS mode was used for PD scoring. The PA imaging with stronger signals at either the wavelengths of 750nm and 830nm (the second and the third part of the screen) was used for PA scoring. The maximal score (0-3) of either synovitis or tenosynovitis/paratenonitis would be adopted as the final score for each joint.

The GSUS, PDUS and PA images were assessed by two radiologists with 2-year experiences in musculoskeletal US, who were blind to the patients’ information and clinical manifestations of the examined joints. When discrepancies of the scores were found between the two radiologists, the inflamed areas for scoring would be re-confirmed and the pseudo-color PD and PA signals would be re-assessed by the two readers along with the US operator, until consensus of each joint was acquired. During the process of imaging evaluation, conflicting results tended to occur in score 2 and 3, as well as in score 1 and 2. We would generally choose the smaller score as the final result after confirming the range of the inflamed regions.

**Supplementary Data S4. The training project for participated radiologists**

A radiologist who had five years of experience in operating US and had one year of musculoskeletal US experience performed the multimodal imaging examination. The operator received a series of training courses and practiced musculoskeletal ultrasound (MSK US) for 20-30 patients a week for about one year. Before initiating the formal clinical study, the pre-examinations of multimodal imaging on healthy volunteers and patients were performed by the operator for about one month, and about 15-20 times a week. After practicing the system for one month, the operator was able to perform the multimodal PA/US imaging examination proficiently with high-quality US and PA images and control the whole examining time in 30 minutes.

Before implementing formal multimodal imaging examinations for RA patients in our medical center, a total of 9 radiologists with 4-6 year-experience of US in our medical center, including the operator and the radiologists who read the images for this study, participated in a training project for MSK US. The training project was comprised of a six-month standard training course and an on-line self-learning application (App). The training courses, which were taught by experts of MSK US, included basic anatomy of joints, pathophysiology of musculoskeletal diseases, imaging presentations of normal and impaired joints, imaging features of classical articular disease, as well as practical operations of MSK US on healthy volunteers and patients with musculoskeletal diseases. The 0-3 scoring method for power-Doppler US images and PA images was further studied in the App, by showing typical images with different scores. After receiving standard trainings, the radiologists also took part in clinical practice of MSK US once a week.

After three months of clinical practice, a test of interpreting US images of RA patients was launched. The test database included PD and PA images of different scores of MCP2, MCP3, PIP2, PIP3, MTP2, MTP5 and wrist joints, with 10 slices for each part. The intraclass correlation coefficients (ICC) with 95% CI was calculated to evaluate the inter-rater variability of the nine raters in both PD and PA scoring. The ICC value was interpreted as follows: poor agreement: ICC <0; slight agreement: 0<ICC<0.20; fair agreement: 0.20<ICC<0.40; moderate agreement: 0.40<ICC<0.60; good agreement: 0.60<ICC<0.80; very good agreement: 0.80<ICC< 1.

Systematic differences among the raters were found to be relevant after analysis of variance (p<0.05) for both PD scoring and PA scoring, and the ICC was regarded as a measure of absolute agreement. The average measures of ICCs and 95% CI were 0.934 (0.905-958) for PD scoring and 0.919 (0.885-0.947) for PA scoring, respectively, indicating that the radiologists reached very good agreement after receiving the training project. The participants also presented good to very good agreement with the standard scoring (κ_1_[PD] = 0.87 [0.75-0.93] [***P*** = 0.040], κ_1_[PA] = 0.81 [0.70-0.89] [***P*** = 0.046]; κ_2_[PD] = 0.85 [0.72-0.91] [***P*** = 0.043], κ_1_[PA] = 0.81 [0.71-0.88] [***P*** = 0.045]; κ_3_[PD] = 0.92 [0.86-1.00] [***P*** = 0.041], κ_3_[PA] = 0.87 [0.79-0.96] [***P*** = 0.043]; κ_4_[PD] = 0.86 [0.76-0.96] [***P*** = 0.041], κ_1_[PA] = 0.82 [0.76-0.88] [***P*** = 0.047]; κ_5_[PD] = 0.83 [0.72-0.88] [***P*** = 0.042], κ_5_[PA] = 0.79 [0.69-0.81] [***P*** = 0.046]; κ_6_[PD] = 0.83 [0.71-0.88] [***P*** = 0.043], κ_6_[PA] = 0.80 [0.72-0.89] [***P*** = 0.046]; κ_7_[PD] = 0.91 [0.82-0.99] [***P*** = 0.040], κ_1_[PA] = 0.86 [0.78-0.91] [***P*** = 0.043]; κ_8_[PD] = 0.86 [0.79-0.94] [***P*** = 0.041], κ_8_[PA] = 0.83 [0.74-0.92] [***P*** = 0.045]; κ_9_[PD] = 0.88 [0.81-0.98] [***P*** = 0.042], κ_9_[PA] = 0.80 [0.70-0.91] [***P*** = 0.048]). The radiologists presented a good performance in scoring PDUS and PA images after the standard training of MSK US.

**Supplementary Data S5. Relative SO_2_ values and SO_2_ subgrouping**

The fourth part of the screen, which was designed as the mapping of oxygenation in pseudo-color was used for SO_2_ measurement. To accurately discriminate the signals representing hyperoxia and hypoxia, the pixels of the integrated signals were quantified as the relative values of SO_2_. By calculating the ratio of the pixels of PA signals in the target areas at the wavelength of 750nm and 830nm, the relative SO_2_ values of the inflamed region were determined. The calculating software was loaded on the ultrasonic system, and the SO_2_ calculating process could be performed through tracing the targeted regions at the SO_2_ interface on the screen by radiologists. After drawing the ROIs, the relative SO_2_ value would be presented on the lower right corner of the screen automatically. The joint with highest PA signals was selected to make the SO_2_ calculations and used as the local SO_2_ value of each patient. Three times of calculations were done for each joint, and a mean value was identified as the representative oxygenation status of the individual patients.

The box plot of the relative SO_2_ values of the patients were presented in Supplementary Figure S1, from which a dichotomic distribution of the values with a gap of approximately 85%-90% could be observed. The patients were divided into the hyperoxic subgroup and the hypoxic subgroup according to the distribution of the relative SO_2_ values. Twelve patients were classified as hyperoxia with the relative SO_2_ value greater than 90%, and nine patients were hypoxia with the value smaller than 85%. The SO2 values of the small joints was 87.5 ± 10.1%.


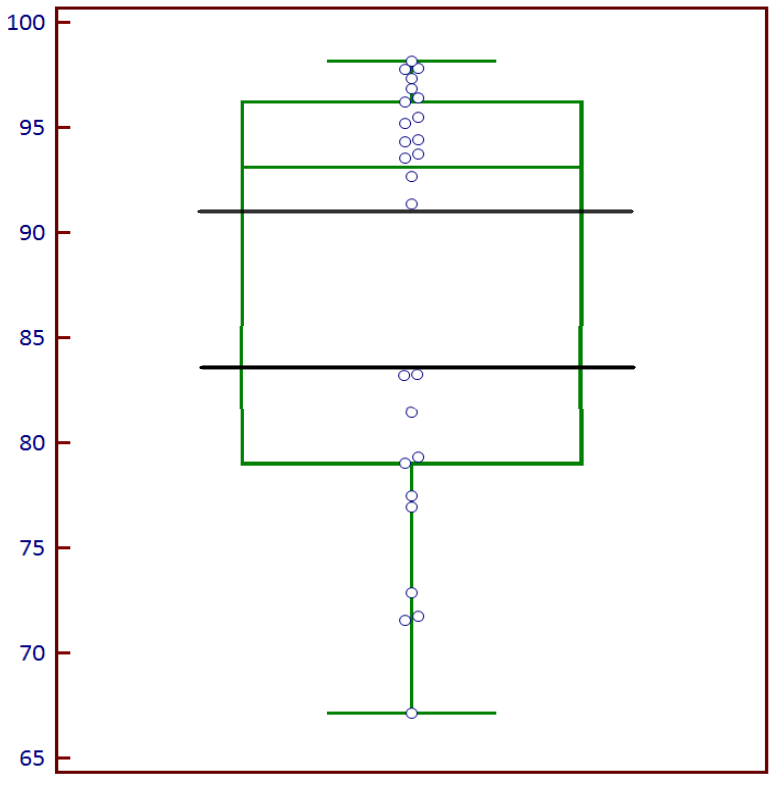


**Supplementary Figure S1.** The box plot of the relative SO2 values of the 21 RA patients (PA-sum > 0). A dichotomic distribution of the 21 values could be observed with a gap of around 85%-90%. The patients with SO2 values greater than 90% were classified as hyperoxia, and the patients with SO2 values smaller than 85% were classified as hypoxia.
